# Agar/β-Cyclodextrin Composite Cryogels for Controlled Release of Aripiprazole

**DOI:** 10.3390/molecules30051051

**Published:** 2025-02-25

**Authors:** Siyka Stoilova, Georgy L. Georgiev, Rositsa Mihaylova, Bistra Kostova, Petar D. Petrov

**Affiliations:** 1 Institute of Polymers, Bulgarian Academy of Sciences, 1113 Sofia, Bulgaria; s_stoilova@polymer.bas.bg (S.S.); ggeorgiev@polymer.bas.bg (G.L.G.); 2 Department of Pharmacology, Pharmacotherapy and Toxicology, Faculty of Pharmacy, Medical University of Sofia, 2 Dunav St., 1000 Sofia, Bulgaria; rmihaylova@pharmfac.mu-sofia.bg; 3 Department of Pharmaceutical Technology and Biopharmacy, Faculty of Pharmacy, Medical University of Sofia, 1000 Sofia, Bulgaria; 4 Centre of Competence “Sustainable Utilization of Bio-Resources and Waste of Medicinal and Aromatic Plants for Innovative Bioactive Products” (BioResources), 1000 Sofia, Bulgaria

**Keywords:** aripiprazole, agar, β-cyclodextrin, cryogels, drug delivery

## Abstract

Aripiprazole (ARZ) is an atypical antipsychotic drug used to treat a variety of mood and psychotic disorders, such as schizophrenia, bipolar disorder, major depressive disorder, autism, and Tourette’s syndrome. Although ARZ offers significant therapeutic benefits, its poor solubility in water requires the development of delivery systems aimed at improving the solubility and bioavailability of the drug. In this work, cryogels based on two natural products—agar and β-cyclodextrin (CD)—were developed and evaluated as a drug delivery system for ARZ. The cryogels were prepared by cryogenic treatment of aqueous solutions of agar and the β-CD/ARZ complex, followed by thawing. The main characteristics of the material, including gel fraction yield, swelling degree, pore volume, elastic properties, and morphology were studied in detail. The release of ARZ from composite cryogels was assessed in two media resembling the pH in stomach and intestine. The system exhibited a pH-dependent release of ARZ, with a slower rate in acidic media (pH 1.2) than in the neutral phosphate buffer (pH 6.8). Under in vitro conditions, the amount of released ARZ over 48 h reached 33%.

## 1. Introduction

Aripiprazole is a third-generation antipsychotic drug. It has a unique pharmacological profile and can be used as a therapeutic tool for major psychiatric disorders [1]. Aripiprazole’s mechanism of action is characterized by its partial agonist activity at dopamine D_2_ and D_3_ and serotonin 5-HT_1A_ receptors, as well as antagonist activity at serotonin 5-HT_2A_ receptors. This mechanism of action leads to successful reduction of positive, negative, and cognitive symptoms of schizophrenia [2]. ARZ can be used for the following indications: schizophrenia, bipolar disorder (manic and mixed episodes), major depressive disorder, irritability associated with autism spectrum disorder, and vocal and motor tics associated with Tourette’s syndrome [3]. The drug is typically tolerated well, despite some common adverse effects such as nausea, dyspepsia, headache, agitation, and motoric activation similar to akathisia [4]. ARZ belongs to class IV of the biopharmaceutical classification system, where the drugs are characterized with low permeability and bioavailability. Its solubility in water is poor (less than 0.3 mgL^−1^). Therefore, extensive efforts have focused on creating delivery technologies and systems to enhance the solubility and thus therapeutic efficiency of ARZ. Ramya et al. enhanced the solubility and dissolution rate of ARZ by formulating a self-micro emulsifying drug delivery system [5]. Silki et al. have developed ARZ-loaded solid lipid nanoparticles (SLNs) using tristearin as solid lipid and Tween 80 and sodium taurocholate as surfactants to prepare SLNs using a microemulsification method [6]. Kumbhar et al. formulated mucoadhesive nanoemulsion of ARZ intended for intranasal delivery to transport the drug directly to the brain [7]. In these studies, the good solubilizing effect has been achieved mainly due to the use of synthetic surfactants and co-surfactants. Synthetic polymeric micelles [8], solid polymer matrixes [9,10,11], and mesoporous silica [12] have also demonstrated a promising dissolution capability and potential to enhance the oral bioavailability of ARZ. Natural products have also been exploited for the elaboration of delivery systems for ARZ. For instance, Al-Dhubiab et al. designed buccoadhesive chitosan films in which nanocrystals of ARZ were incorporated [13]. The developed nanocrystal-loaded buccal films have demonstrated good potential to provide a faster availability of ARZ, which is considered a promising option for patients with schizophrenia, especially for geriatric patients. In another work, Awais et al. have prepared binary and ternary inclusion complexes of ARZ with methyl-β-cyclodextrin (MβCD) and L-Arginine (LA), resulting in enhanced solubility and decreased crystallinity of the drug [14]. The authors claimed that the method of preparation (lyophilization) and the specific drug-to-polymer and amino acid ratio were critical for achieving high drug solubility and stability.

Cyclodextrins (CDs) and their derivatives have drawn considerable attention in science and technology due to their high availability, large-scale production, and non-toxic nature [15]. The three most studied native CDs are α-CD, β-CD, and γ-CD, which consist of six, seven, and eight α -(1,4)-linked glycosyl units, respectively. The free hydroxyl groups on the exterior of CDs makes them hydrophilic, while the oxygen atoms in the glycosidic bonds and the hydrogen atoms within the cavity create a hydrophobic internal environment. This unique structure enables CDs to dissolve poorly soluble compounds (including drug molecules) in aqueous media by host–guest interactions [16]. The potential of CDs for use in drug delivery has been demonstrated by many studies [17,18,19,20,21,22] and recently reviewed [23]. Due to several beneficial characteristics, such as low cost, proper cavity size, great complexation ability, and high stability constant, β-CD and its derivatives have been widely used in this field. Concerning ARZ delivery, novel carriers based on N,N-dimethylacrylamide and β-cyclodextrin triacrylate were designed in the form of cryogels [24] and nanoscopic gels [25]. The two systems exhibited pronounced solubilizing ability for ARZ and a sustained release pattern. The encouraging results from these studies motivated our team to develop cryogels from natural products as a drug delivery system for ARZ. 

Agar is a polysaccharide produced by many red algae (Rhodophuceae), such as Gelidiaceae, Gracilariaceae, Gelidiellaceae, and Pterocladiaceae [26]. Agar contains mainly agarose and a smaller amount of agaropectin [27]. Agar has some specific characteristics which define its wide use: good rheological properties, good compatibility with other polysaccharides, and low cost. It has a relatively low melting point (85 °C) and a low solidifying temperature (generally around 32–45 °C) [28]. These properties, in combination with its natural origin, make agar attractive for use in the food, biomedical, and chemical industries. One of the main applications of agar is in microbiology as a gelling agent for cultural media [29]. Due to its large molecular structure and limited bioavailability in the digestive system, agar exhibits minimal bioactivity [30]. When administered orally, agar is fermented and metabolized by gut microbiota in the low gastrointestinal tract [31].

Cryogels are super-macroporous hydrogels formed at sub-zero temperatures by freezing and thawing a solution containing monomer or polymer precursors [32]. Cryogels possess a sponge-like structure with an interconnected network of macropores that makes these materials attractive for application in the fields of biomedicine, biotechnology, and pharmaceuticals [33,34]. Cryogels can be used for biomolecule immobilization, target molecule capture, controlled drug delivery, tissue engineering, bioreactors, cell separation, and as scaffolds [35]. For such purposes, natural materials are preferred to synthetic ones because of their biocompatibility and biodegradability, lack of toxicity, better interaction with surrounding tissues, etc. The present work describes the development of a novel cryogel carrier for ARZ fabricated from agar and β-CD. Both agar and β-CD are low-cost natural products that are biocompatible and therefore suitable for developing drug delivery systems. In particular, β-CD is used to increase the solubility of the hydrophobic ARZ in water and modulate drug release. To the best of our knowledge, there are no reports in the literature on cryogel systems designed for delivery of ARZ based on poly/oligosaccharides such as agar and β-CD. First, a complex of β-CD and ARZ was formed in an aqueous medium to provide a water-soluble form of the drug, which was subsequently embedded into the agar matrix. The principle physico-chemical characteristics of the system, as well as the release profile of ARZ in two media resembling the pH in stomach and intestine, were investigated in detail.

## 2. Results

### 2.1. Synthesis of Cryogel

**Figure 1:**
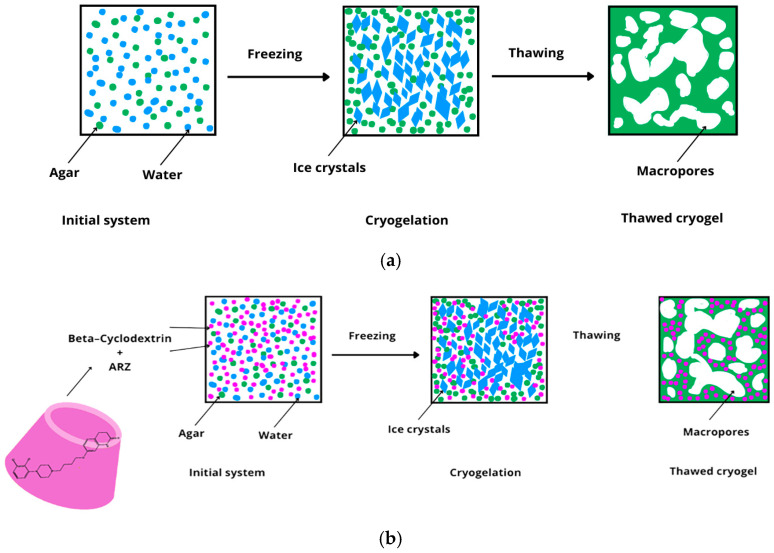
Sketch of the fabrication of: (**a**) pure agar cryogels, and (**b**) ARZ-loaded agar/β-CD cryogels.

In the first stage of our study, pure agar cryogels were prepared and investigated to find a polymer matrix with appropriate physical and mechanical properties which could be exploited as a platform for developing carriers for ARZ. Six series of cryogels were synthesized by a facile procedure involving dissolution of agar (0.5, 1, 2, 3, 4, and 5 w/w%) in warm water (90 °C) and subsequent freezing of the polymer solution at −20 °C. The frozen samples (disks with a diameter of 20 mm and a thickness of 5 mm) were kept at −20 °C for two h and then thawed (Figure 1a). For some analyses, the cryogels were immersed in water for 3 days and then lyophilized. Next, ARZ-loaded (or blank) composite agar/β-CD cryogels were obtained by a similar procedure where the aqueous solutions of agar and β-CD-ARZ complex were blended immediately before freezing (Figure 1b).

### 2.2. Gel Fraction Yield and Swelling Degree of Agar Cryogels

**Figure 2:**
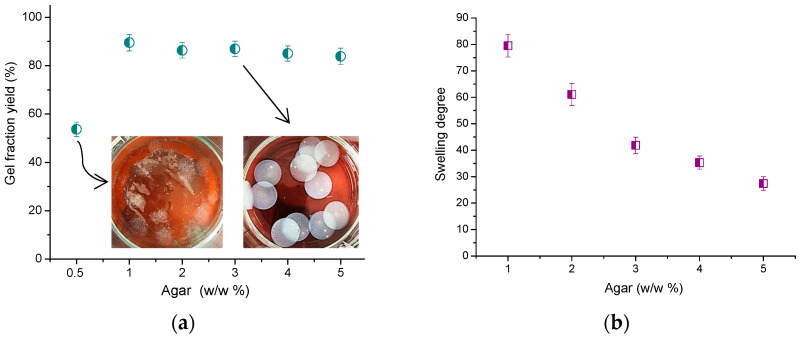
(**a**) Gel fraction yield, and (**b**) swelling degree of different agar cryogels. Insets:defragmented cryogel discs from 0.5 w/w% agar solution (**left**); and monolithic cryogel discs from 3 w/w% agar solution (**right**).

Overall, the agar cryogels synthesized by cryotropic gelation were compact, opalescent soft materials. Only the gel obtained from the 0.5 w/w% polymer solution was weak and disintegrated upon immersion in water (inset in Figure 2a). The gel fraction (GF) yield of agar cryogels were determined gravimetrically. This experiment aimed to establish what fraction of the initial polymer was incorporated into the gel at the reported experimental conditions. For this purpose, the disks were kept for 3 days in distilled water, which was replaced several times to extract the water-soluble polymer fraction. As seen in Figure 2a, the GF yield of all samples was relatively high (up to 89%), except for the gel formed from 0.5 w/w% polymer solution. These results revealed that most of the polymer molecules were incorporated into a physical network, which maintained the gel structure stable at room temperature even in excess water. Only a small portion (*ca.* 10%) of agar was dissolved at such conditions. The swelling ability of cryogels in pure water was assessed as well. As expected, the dimensions and mass of the prepared agar cryogels (in swollen state) were similar for the five compositions studied. In addition, we did not observe a notable change to those parameters for all samples after soaking in water for 3 days. It was found that the mass of water in the five series of gels varied from 0.74 to 1.23 g, while the mass of the corresponding freeze-dried gels was between 0.0094 and 0.044 g (Table 1). These results indicate a predominant water content in the material, as the difference between the mass of water and polymer reached 25–80 times, depending on the composition. The calculations of the swelling degree (SD) of the cryogels revealed a gradual decrease in this parameter, with increasing polymer content in the initial aqueous solution and cryogel (Figure 2b). The significant change in SD value is most likely due to the notable increase in the mass of agar in the gel (>4.5 times for the gel formulations of 1 and 5 w/w%), and not the amount of uptaken water, which was only 1.7 times higher (see Equation (2)).

### 2.3. Morphology and Pore Volume

**Figure 3:**
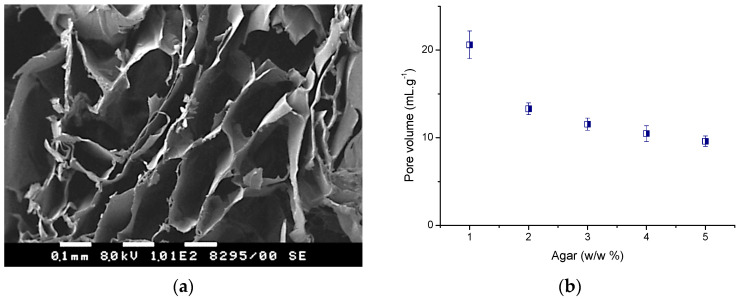
(**a**) Selected SEM micrograph of agar cryogel obtained by cryogelation of 3 w/w% polymer solution, and (**b**) pore volume of different agar cryogels, calculated by using methanol as a non-solvent.

The morphology of agar cryogels were investigated by scanning electron microscopy (SEM) with a focus on cross-sections of freeze-dried samples. We observed an open porous structure for all gels, as illustrated in Figure 3a. Most of the pores were characterized by an irregular shape and the pore size varied from tens to hundreds of microns. The pores were surrounded by much thinner walls (several microns) representing the polymer matrix. Unfortunately, the highly heterogeneous structure of materials makes it unfeasible to compare different compositions in terms of pore diameter and size distribution.

Another important parameter of cryogels is their total pore volume. It was determined for freeze-dried samples, which were immersed in a nonsolvent such as methanol, following a procedure described elsewhere [36]. The poor solvent only penetrated the pores without causing swelling of the cryogel walls (polymer network). It is evident that with increasing the concentration of agar in polymer solution the pore volume of cryogel decreased (Figure 3b). The cryogel fabricated from the most dilute agar solution (1 w/w%) exhibited a pore volume two times larger than the most concentrated sample (5 w/w%). Nevertheless, except for the lowest concentration, the pore volume of the other four cryogels varied in a relatively small range, between 13 and 10 mL g^−1^.

### 2.4. Dynamic Rheological Measurements

**Figure 4:**
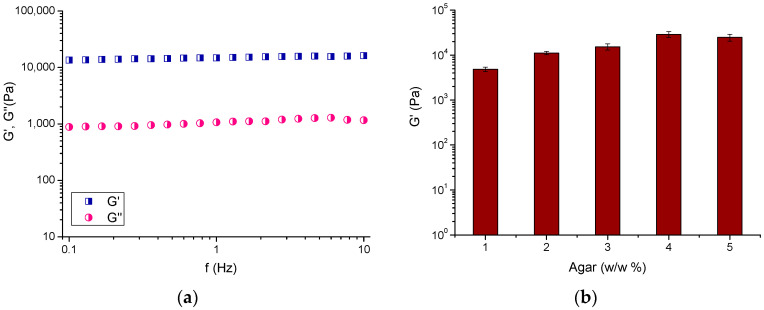
(**a**) The elastic (G′) and loss (G″) moduli of an agar cryogel obtained by cryogelation of 3 w/w% polymer solution (single measurement), and (**b**) G′ of agar cryogels of different composition determined at 1 Hz. Each sample was measured in triplicate and the error bars are the standard deviation.

The elastic properties of cryogel materials were studied by oscillation frequency sweep tests. The measurements of elastic (G′) and loss (G″) moduli were performed at a constant shear deformation (γ = 0.001) in the 0.1–10 Hz frequency range. Such a low value of γ belongs to the viscoelastic range (determined by the amplitude sweep test) and was chosen to avoid any destruction of the sample structure by the applied stress. The rheological measurements of agar cryogels showed that these are highly elastic materials. First, the values of G′ (elastic portion) were more than one order of magnitude larger than G″ (viscous portion) in the whole frequency region (Figure 4a). This means that the deformation energy is stored in the material, by extending and stretching the internal superstructures, without significant loss. The calculated loss factor (tan δ = G″/G′) was approximately 0.07 ± 0.002, which is much closer to the value of an ideally elastic material (tan δ 0.01) than to an ideal fluid (tan δ 100) [37]. Another important finding was that the absolute value of G’ approached and exceeded 10 kPa, depending on the gel composition (Figure 4b). Thus, the developed agar cryogels possess elastic properties similar to conventional agar hydrogels [38].

### 2.5. Solubilization of ARZ in Water by β-CD

**Figure 5:**
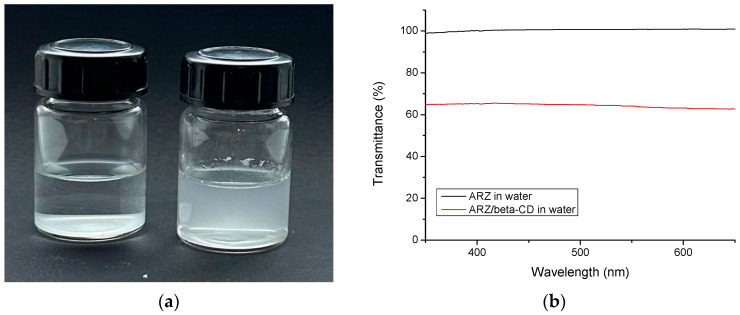
(**a**) Digital picture of pure ARZ (**left**) and β-CD-ARZ complex (**right**) in water, and (**b**) turbidity curves of the same samples, examined 6 h after preparation.

The solubility of ARZ in water was enhanced with the aid of β-CD, taking advantage of the affinity of ARZ molecules for the hydrophobic cavity of β-CD, also known as the host–guest complexation phenomenon [24,25]. A molar ratio of β-CD/ARZ = 2 was used in this study because in previously published works, describing in detail the host–guest interaction of the two molecules, such a ratio was considered appropriate for stable complex formation [38,39]. Initially, an aqueous solution of β-CD was blended with a methanol solution of ARZ at a β-CD/ARZ mass ratio 5 (molar ratio 2), and then the organic solvent was removed using a rotary vacuum evaporator to yield an aqueous dispersion of β-CD-ARZ complex. To confirm the solubilizing effect of β-CD, we performed a control experiment by repeating the preparation procedure described above but without adding β-CD. The two samples were stored for 6 h at room temperature and then examined. As evident from the digital picture in Figure 5a, ARZ precipitated in water when β-CD was not present in the media. In contrast, no precipitate was observed in the dispersion with β-CD. Turbidity measurements of the two systems with a UV-Vis spectrophotometer showed nearly 100% transmittance for pure ARZ in water and 65% transmittance for the β-CD-ARZ complex dissolved in water (Figure 5b).

### 2.6. Blank and ARZ-Loaded Agar/β-CD Cryogels

**Figure 6:**
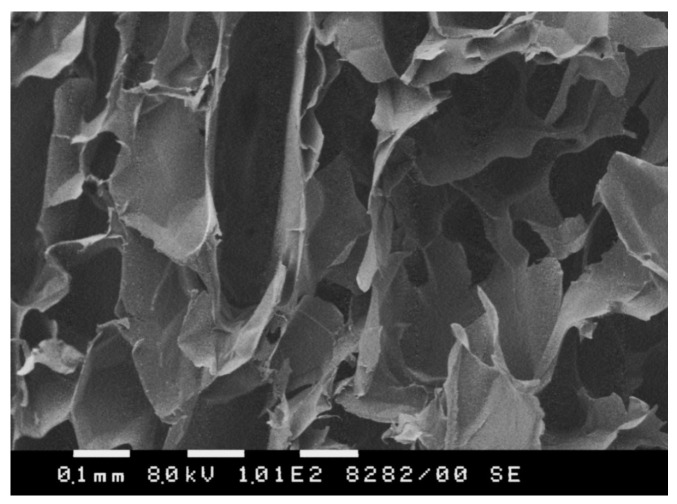
Selected SEM micrograph of an agar/β-CD cryogel, obtained by cryogelation of 3 w/w% polymer solution.

Agar/β-CD cryogels with and without ARZ were obtained by the cryogelation procedure used for the synthesis of pure agar cryogels. In this case, a preformed blend of agar and β-CD-ARZ complex (or agar and β-CD) in water were frozen and thawed (Figure 1b). The concentration of agar in the solution was 3 w/w% and the mass ratio of agar/β-CD/ARZ was 30:1:0.2. The cryogel without ARZ was prepared to gain insight into the influence of β-CD on the physico-mechanical properties of the material. The incorporation of 3.33 mass% oligosaccharide into the agar matrix did not change significantly the GF yield and swelling degree of cryogel but increased the elastic modulus of the cryogel (Table 2). The morphology of the composite gel was identical to that of the pure agar cryogel, as confirmed by SEM (Figure 6). This analysis revealed the typical heterogeneous structure of such materials, characterized by very large, interconnected pores.

**Figure 7:**
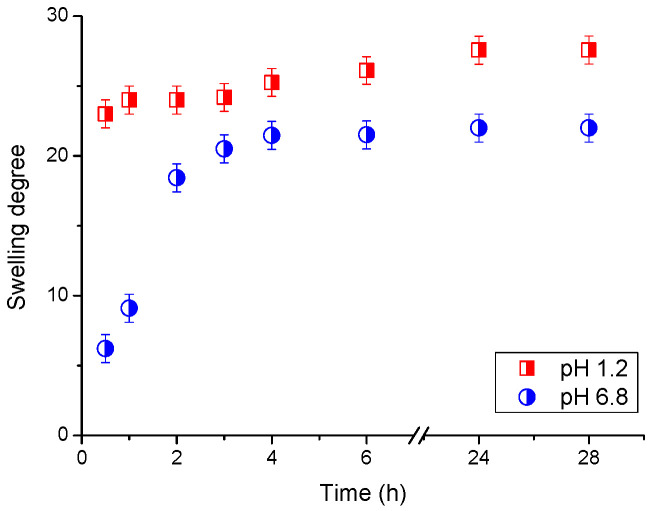
Swelling degree as a function of time of an agar-β-CD cryogel in hydrochloric acid solution with pH 1.2 and phosphate buffer with pH 6.8.

Considering the potential application of cryogels for peroral application, we studied the swelling behaviour of an agar/β-CD cryogel in a hydrochloric acid solution with pH 1.2 and a phosphate buffer with pH 6.8. The pH of these two fluids resembles the pH in stomach and intestine, respectively. The results from this test showed that the swelling of cryogel is pH-dependant and the freeze-dried material swells faster in acidic media than in phosphate buffer (Figure 7). This finding can be explained by the capillary action effect observed when liquids are drawn into porous materials. Since the diameter of the pores is small enough, a combination of surface tension (which is caused by cohesion in the liquid) and adhesion forces between the liquid and the cryogel wall act to propel the liquid. Apparently, the surface tension of hydrochloric acid is lower than that of a neutral buffer, which helps the liquid to enter and fill the pores of the gel more quickly. The swelling degree of the agar/β-CD cryogel, calculated after 28 h incubation at pH 1.2 and 6.8, was 28 and 22, respectively. It seems that intermolecular interactions between the liquid and polymer matrix are stronger in acidic media than in a neutral buffer.

### 2.7. Drug Release Studies

**Figure 8:**
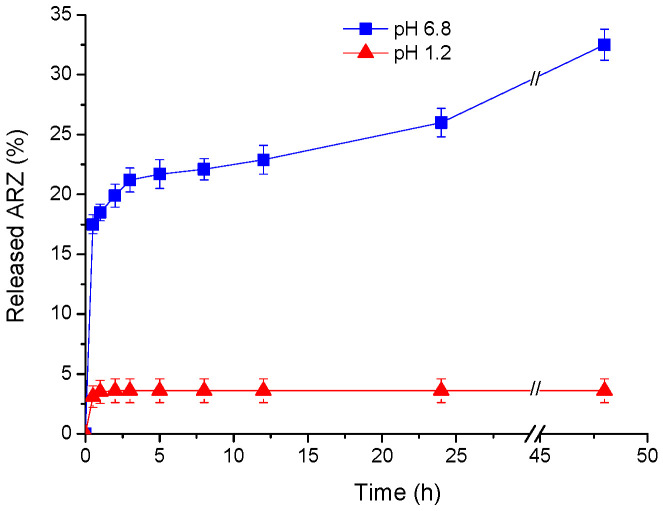
In vitro release of aripiprazole from agar-β-CD cryogel at pH 1.2 and 6.8.

The in vitro release of ARZ from the cryogel matrix in hydrochloric acid solution (pH 1.2) and phosphate buffer (pH 6.8) was studied spectrophotometrically at a temperature of 37 ± 0.5 °C. The release profiles are shown in Figure 8. Apparently, the release process was strongly influenced by the pH of the medium. The released ARZ at pH 1.2 within the first 2 h was only 4% and it remained unchanged until the end of the test period. A much higher amount of the drug was released in the media with pH 6.8. We observed an initial fast release of approximately 17% of ARZ for 30 min, followed by a sustained release. Finally, 33% of the drug was released over 48 h.

### 2.8. MTT Cell Viability Assay

**Figure 9:**
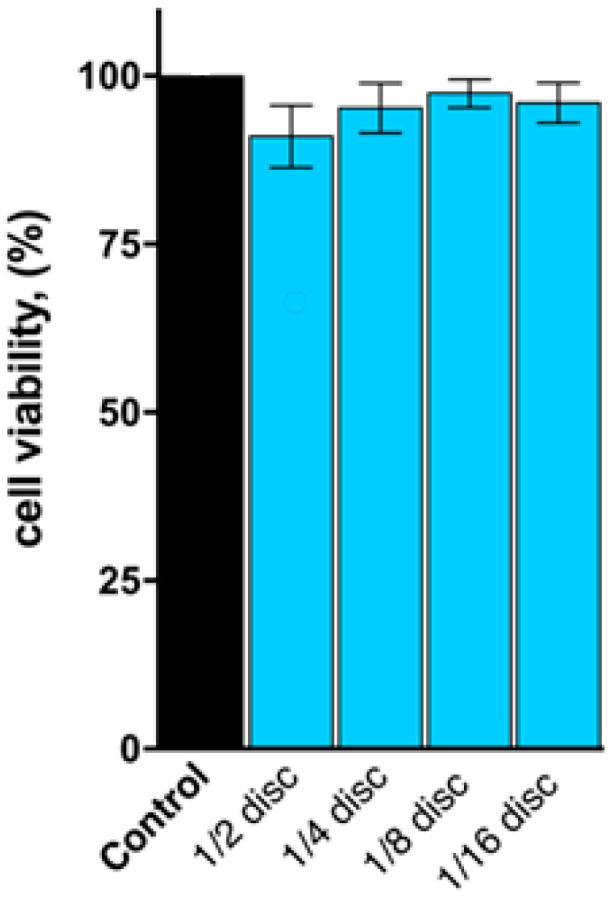
Cell viability of NEURO-2A cells in the presence of different portions of agar cryogel (3 w/w%) against a negative untreated control. All experiments were run in triplicate and data are expressed as a mean ± SD. Statistical analysis of the data was performed using a one-way ANOVA and values of *p* ≤ 0.01 were considered statistically significant.

The in vitro biocompatibility of blank agar cryogel was assessed against neuronal NEURO-2A cells, after a 72 h incubation period. As evident from Figure 9, treating cells with four different portions of the carrier (pieces ranging in size from 1/16 to 1/2 of the as-prepared disc) had no statistically significant impact on the viability and proliferation capacity of the neuroblast cells. In addition, no morphological changes in cell adherence and appearance regarding size, form, or membrane integrity were detected by microscopic studies.

## 3. Discussion

The gelling ability of agar is due to the presence of agarose in its composition, as described elsewhere [40]. Agarose is a linear carbohydrate polymer, with a repeating unit made of D-galactose and 3,6-anhydro-L-galactopyranose, which is water insoluble in cold water but dissolves at a temperature near the boiling point of water. At such high temperature, agarose molecules have a random coil conformation. However, at a lower temperature (30–40 °C), agarose macrochains, because of hydrogen bonding and electrostatic interaction, form helical structures arranged into a three-dimensional network with entrapped water (hydrogel) [41]. In our case, the agar solutions were quickly placed in a freezer (−20 °C), and therefore we assume that the gelation process was completed at sub-zero temperatures. The cryogenic treatment was the key factor for the formation of a super-macroporous structure of materials. The frozen aqueous polymer systems are characterized by two phases: a solid phase consisting of pure ice crystals and a liquid microphase containing dissolved polymer molecules [42]. Thus, a physical network was formed in the liquid microphase that surrounds the forming crystals. The ice crystals acted as a porogen, and after thawing the system, the resulting super-macroporous gel comprised interconnected large pores. Since various substances can easily pass through such a structure, agar cryogels can be considered a promising platform for developing drug delivery systems. The concentration of the initial polymer solution affected the GF yield and physico-mechanical properties of the gels, respectively. Highly elastic (>10 kPa) monolithic cryogels were formed at agar concentrations ≥1 w/w%. It should be mentioned that above 4 w/w% agar concentration, the solution became highly viscous and difficult to handle. Therefore, the composite agar/β-CD cryogels were fabricated from a 3 w/w% polymer solution. As mentioned above, β-CD was used to enhance the solubility of ARZ in water by forming a water-soluble complex. It was demonstrated that the pure hydrophobic drug (dissolved in methanol) quickly agglomerates in aqueous media and a pronounced phase separation occurs. Consequently, a homogeneous system cannot be obtained upon mixing with the polymer solution. On the other hand, the β-CD/ARZ complex in water was stable enough to complete the blending with the agar solution and to perform the cryogelation. Considering the mechanism of this process, we suggest that drug-loaded oligosaccharide molecules were embedded mainly into the agar matrix. The calculated values for the swelling degree and pore volume of cryogels were concentration dependant and decreased with increasing the amount of agar. However, the dimensions of cryogels obtained from polymer solutions of different concentration (1–5 w/w%) were identical, i.e., no significant expansion of the disks was observed for all compositions. This means that the fraction of uptaken free water, located in the pores of the material, is much higher than that of the physically bound water. In other words, the helical structures building the polymer network do not favour adsorption of a large volume of water, and the apparent high difference in the swelling degree (for example, between 1 and 5 w/w% gels) is more likely due to the fivefold increase in the mass of the polymer than the mass of water (Table 1, Equation (2)). Similarly, incorporating more polymer into the gel afforded materials with high elastic modulus. Embedding rigid β-CD rings into the polymer matrix imparted additional strength to the gels. Since β-CD-ARZ complex was added to the polymer solution during the preparation of the cryogel, it can be assumed that the entire amount of the drug was incorporated into the gel carrier. Moreover, considering the specific peculiarities of the cryogenic process [40], we suppose that β-CD-ARZ complex was entrapped predominantly within the agar matrix, building the cryogel walls. In other words, the system consists of a dense polymer matrix containing ARZ, surrounding very large, interconnected pores filled with free water.

As is evident from Figure 7, the freeze-dried cryogels swell faster at pH 1.2 than at pH 6.8, and we supposed that the release of ARZ in the hydrochloric acid solution would be faster. However, the in vitro release profiles of ARZ showed the opposite trend. In a previous work, we reported similar results for a poly(N,N-dimethyl acrylamide)-based drug delivery system containing β-CD/ARZ complex [24]. These facts lead us to believe that the complex plays a key role in the drug release process. ARZ is weakly alkaline with pH-dependant solubility [43], and one reason for the low rate of release in the hydrochloric acid solution could be the high strength of the host–guest complex. Obviously, the complex is much more stable in the medium with low pH, making the release of drug molecules more difficult. In contrast, in phosphate buffer (pH 6.8) the β-CD-ARZ complex is weaker, and the drug release is facilitated. In these conditions, the amount of released ARZ for 48 h reached 33%, which can be considered a high value, taking into account the limited solubility of ARZ in a neutral physiological medium. The drug release tests and biocompatibility studies demonstrated that the developed system is suitable for peroral application, because the agar matrix is not cytotoxic and ARZ will be kept by the carrier passing through the stomach and will be released in the intestine.

## 4. Materials and Methods

### 4.1. Materials

Agar, methanol, and β-CD were purchased from Sigma-Aldrich (FOT, Sofia, Bulgaria) and used as received. Aripiprazole was purchased from Fengchengroup (Qingdao, China).

### 4.2. Methods

#### 4.2.1. Synthesis of Cryogel

Six series of cryogels were prepared from aqueous agar solution with different concentrations (0.5, 1, 2, 3, 4, and 5 w/w%). The corresponding amount of agar was added to a beaker containing 50 mL of water. The mixture was heated in an oil bath to 90 °C with constant stirring. After the agar was completely dissolved, the heating was stopped and the solution was allowed to cool down to about 50 °C and quickly dispensed into 12 Teflon moulds, containing 1 mL each. The samples were frozen at −20 °C in a freezer and kept for 2 h. Next, the gels were thawed and immersed in an excess of water for 3 days. The water was exchanged for a fresh quantity 4 times.

#### 4.2.2. Calculation of Gel Fraction Yield and Swelling Degree

The swollen samples were weighed, freeze dried, and weighed again. The gel fraction yield and swelling degree of the cryogels were determined gravimetrically using the following equations [24]:

GF yield (%) = (mass of freeze dried sample)/(initial mass of polymer) × 100(1)

SD = mass of swollen sample/mass of freeze-dried sample(2)

#### 4.2.3. Pore Volume

The total pore volume (Vp) was determined by immersing the freeze-dried cryogels in an excess of methanol until constant mass was reached at room temperature. The samples were then weighed. The total pore volume was calculated using the following equation:

(3) $$Vp\;({mL\;g}^{-1})=\frac{m_{p-}m_{dry}}{d_{p}m_{dry}}$$

where: Vp is the total pore volume, m_p_ is the mass of the sample in the poor solvent, m_dry_ is the mass of the dried sample, d_p_ is the density of methanol (0.792 g·mL^−1^).

#### 4.2.4. Dynamic Rheological Measurements

Dynamic rheological measurements of the cryogels were carried out with a HaakeRheoStress600 rheometer (Thermo Fisher Scientific, Waltham, MA, USA) equipped with a parallel plate geometry (20 mm diameter) and a thermoelectric Peltier controller. The variation of elastic (G′) and loss (G″) moduli as a function of the frequency were measured in the 0.01–10 Hz range at 25 °C in CD-mode. The shear deformation (γ) was 0.001, which is inside the linear viscoelastic regime. Three runs of each sample were conducted. The phase shift δ between the stress and deformation (time lag between the preset and the resulting sinusoidal oscillation) was determined for each measuring point. G′ and G″ were calculated using the following equations:

G′ (Pa) = G*·cos δ(4)

G″ (Pa) = G*·sin δ(5)

G* is the complex modulus, calculated by setting the stress amplitude and measuring the deformation amplitude.

#### 4.2.5. Scanning Electron Microscopy

The cross sections of freeze-dried agar cryogels were observed by a JEOL 6390 scanning electron microscope (JEOL, Ltd., Tokyo, Japan), working at an accelerating potential of 8.00 kV. The specimens were fractured just before measurements, fixed on a glass substrate, and coated with gold for 60 s.

#### 4.2.6. Drug Loading

A solution of β-CD with a concentration of 1 g·L^−1^ was prepared by dissolving 25 mg of the oligosaccharides in 25 mL of distilled water. Separately, 5 mg of ARZ was dissolved in 10 mL of methanol, giving a concentration of 0.5 g·L^−1^. The two solutions were mixed in a bottom round flask, and then methanol was evaporated on a rotary vacuum evaporator to obtain 25 mL of an aqueous colloidal solution comprising β-CD (25 mg) and ARZ (5 mg). Next, 0.75 g of agar was added to the remaining solution and a 3 w/w% composite cryogel was prepared following the synthetic conditions described for the pure agar gels. The masses of agar, β-CD, and ARZ in one cryogel disk were 30, 1, and 0.2 mg, respectively.

#### 4.2.7. Drug Release Studies

The in vitro release profiles of ARZ were obtained by using a water shaking bath (IKASH—B 20, Staufen, Germany). The drug-loaded cryogels were tested in 50 mL of hydrochloride acid and phosphate buffer solutions with pH 1.2 and pH 6.8, respectively. The experiments were carried out at a constant agitation speed of 50 rpm at 37 ± 0.5 °C. At specific time intervals, 2 mL samples were withdrawn for analysis. After each sampling, the extracted volume was replaced with an equal amount of fresh medium. ARZ concentration in the collected samples was determined by recording the absorbance at 220 nm with an Evolution 300 UV–Vis Spectrophotometer (Thermo Fisher Scientific, Lenexa, KS, USA). The drug release calculations were based on standard calibration curves. The relationship between ARZ absorbance and ARZ concentration was plotted for the particular pH media in the 1–40 µg·mL^−1^ range and the corresponding equations, describing the linear relationship of the curves: (pH 6.8) y = 0.00845 + 0.08156·x, with a correlation coefficient R = 0.9995, and: (pH 1.2) y = 0.02261 + 0.14744·x, with a correlation coefficient R = 0.9993, were used for the calculation.

#### 4.2.8. MTT Cell Viability Assay

The cytotoxicity of agar cryogels (prepared from 3 w/w% polymer solution) was assessed against NEURO-2A mouse neuroblast cells using a standard MTT-based colorimetric assay. According to the protocol, exponential-phased cells were harvested and seeded (100 μL/well) in 6-well plates at the appropriate density. After a 24 h incubation, cells were treated with four different portions of the disc-shaped cryogel (pieces with dimensions equal to: 1/2, 1/4, 1/8, and 1/16 of the disk). Following a 72 h exposure, filter sterilized MTT substrate solution (5 mg/ml in PBS) was added to each well of the culture plate. A further 24 h incubation allowed the reduction of the yellow MTT reagent into purple formazan crystals in metabolically active viable cells, which were dissolved in isopropyl alcohol solution containing 5% formic acid prior to absorbance measurements at 550 nm. Collected absorbance values were blanked against MTT and isopropanol solution and normalized to the mean value of the untreated control (100% cell viability). The obtained data were fitted to “concentration-effect” curves and analyzed by means of non-linear regression in GraphPad Prism 8.0 software.

## 5. Conclusions

Highly elastic (≥10 kPa), super-macroporous agar/β-CD cryogel carriers for ARZ were developed by a facile procedure based on cryogenic treatment of aqueous solutions of agar and β-CD/ARZ complex. Agar was successfully dissolved in water by heating to 90 °C and then blended with the water-soluble form of ARZ. The gelation process was completed at sub-zero temperatures to afford a drug-loaded composite cryogel. Preliminary studies focused on pure agar systems revealed that cryogel carriers fabricated from 3 w/w% agar solution possess appropriate physical and mechanical properties for developing drug delivery systems for ARZ. The use of β-CD to enhance the solubility of ARZ in water played a key role in preparing a homogeneous mixture prior to cryogelation. The β-CD/ARZ complex also has a notable effect on the release rate of the drug in acidic and neutral media. As a result, it is expected that ARZ will be kept by the carrier when passing through the stomach (2–4 h) and will be released in the intestine. Thus, the developed system can be considered suitable for delivery of ARZ by peroral administration. The method for preparing composite cryogel carriers is not limited to agar and can be applied to other natural or synthetic hydrophilic polymers capable of physical or chemical crosslinking at sub-zero temperatures. Meanwhile, the agar/β-CD carrier can be used to deliver a variety of hydrophobic drugs that form host–guest complexes with β-CD.

## Figures and Tables

**Table 1:** Mass of freeze-dried cryogels and water in the swollen material, obtained by cryogelation of agar solutions of different concentration.

Sample	Mass of Freeze-Dried Gel (g)	Mass of Water in the Gel (g)
Agar 1 w/w%	0.0094 ± 0.00017	0.7380 ± 0.0174
Agar 2 w/w%	0.0181 ± 0.00018	1.0903 ± 0.0180
Agar 3 w/w%	0.0274 ± 0.00043	1.1256 ± 0.0165
Agar 4 w/w%	0.0357 ± 0.00123	1.2264 ± 0.0192
Agar 5 w/w%	0.0440 ± 0.00050	1.1684 ± 0.0199

**Table 2:** Main characteristics of a pure agar cryogel and a composite agar/β-CD cryogel obtained by cryogelation of 3 w/w% polymer solution.

Sample	Gel Fraction Yield (%)	Swelling Degree	Elastic Modulus (Pa)
Agar	87 ± 2	42 ± 2	16,590 ± 2157
Agar/β-CD	88 ± 2	48 ± 3	23,527 ± 1969

## Data Availability

The data presented in this study are openly available in the article.

## Abbreviations

The following abbreviations are used in this manuscript:

ARZ	Aripiprazole
CD	Cyclodextrin
GF	Gel fraction
SD	Swelling degree
